# Nanoporous Copper Fabricated by Mechanically Rubbing the Surface of the Amorphous Alloy

**DOI:** 10.3390/ma18071529

**Published:** 2025-03-28

**Authors:** Lixin Wang, Yuanxiang Zhang, Chenyang Zhang, Jian Kang, Zhenlei Li, Guo Yuan

**Affiliations:** 1 The State Key Laboratory of Digital Steel, Northeastern University, Shenyang 110819, China; 1810248@stu.neu.edu.cn (L.W.); kangjian@ral.neu.edu.cn (J.K.); lizhenlei@ral.neu.edu.cn (Z.L.); yuanguoneu@163.com (G.Y.); 2 Metallurgical College, Northeastern University, Shenyang 110819, China; zhangcy@smm.neu.edu.cn

**Keywords:** amorphous alloy, wear mechanism, roughness, dealloying, nanoporous copper, crack

## Abstract

A surface treatment of amorphous alloy was conducted using reciprocating friction, and precursors with varying degrees of surface roughness were selectively etched to form a three-dimensional nanoporous structure with interconnected networks. The wear behavior induced by friction facilitates dealloying to different extents. While altering the surface roughness of the amorphous alloy, this method preserves its unique structure and maintains the advantages of the precursor in preparing nanoporous materials (NPMs). Under identical dealloying conditions, the thickness of the nanoporous copper layer on the rougher surface (with a surface roughness of approximately 0.808) is significantly greater than that on the smoother surface (with a surface roughness of approximately 0.002), and this disparity increases over time. The findings indicate that friction-induced changes in surface roughness play a crucial role in the preparation of nanoporous copper via dealloying. Modifying the surface roughness through friction can enhance the dealloying process, improve the adhesion between the nanoporous copper (NP-Cu) layer and the amorphous matrix, and mitigate crack propagation during NP-Cu formation and under stress. Selecting an appropriate level of roughness can enhance the long-term stability of NP-Cu.

## 1. Introduction

Nanoporous metal (NPM) synthesized via dealloying exhibits a distinctive three-dimensional interconnected network structure and a high specific surface area, rendering it highly promising for applications across various fields [1,2,3]. Notably, nanoporous copper (NP-Cu) has garnered significant attention in NPM fabrication due to its low material cost, superior electrical conductivity, and exceptional catalytic activity [4,5]. Dealloying has emerged as the predominant method for producing nanoporous copper, owing to its simplicity and tunable pore size [6,7,8]. The choice of dealloying precursor significantly influences the preparation of nanoporous copper. Factors such as elemental composition, phase structure, and microstructure affect dealloying kinetics and corrosion behavior, thereby impacting the structural characteristics of nanoporous copper [9,10,11]. Moreover, the surface treatment of precursors plays a crucial role in enhancing material properties by eliminating surface defects and impurities, thus improving mechanical strength and stability [12,13]. Researchers have demonstrated that surface treatments can enhance the mechanical properties and stability of precursors, which in turn affects the quality of nanoporous copper produced through dealloying. For instance, CHEN et al. [14] developed a novel core–shell-structured nanoporous copper with enhanced sensitivity for glucose detection. Surface friction, as a surface treatment technique, not only modifies surface roughness but also profoundly influences material properties [15,16]. However, current research predominantly focuses on the detrimental effects of friction on metal materials, with limited exploration of its beneficial impacts. Harnessing these beneficial effects could provide new insights into optimizing material performance in diverse service environments.

In this study, an amorphous alloy primarily composed of copper was selected as the precursor material. The surface roughness of the amorphous precursor was modified through rubbing. This research investigates the impact of altered surface roughness on the structure and mechanical behavior of nanoporous copper (NP-Cu) produced via dealloying. Additionally, it provides a comprehensive analysis of the intrinsic relationship between surface roughness and the structural characteristics of NP-Cu, offering valuable insights and effective recommendations for addressing macroscopic fragility and slow natural dealloying in NP-Cu.

## 2. Experiments and Methods

Compared to crystalline metals, amorphous alloys exhibit significant advantages in the dealloying process for the preparation of nanoporous metals (NPMs) [17,18]. This method effectively mitigates the structural damage caused by grain boundaries and segregation. The Zr_48.65_Cu_43.24_Al_8.11_ amorphous alloy demonstrates excellent glass-forming ability and superior mechanical properties. Moreover, the higher reactivity of Zr and Al compared to Cu makes this composition suitable for dealloying, allowing for the successful preparation of nanoporous copper (NP-Cu) [19].

High-purity raw materials, including Zr (99.6%), Cu (99.95%), and Al (99.99%), were used to prepare a molten alloy under an argon protective atmosphere. The alloy was held at 1373 K for ten minutes to ensure complete melting and homogenization. Subsequently, the molten alloy was transferred to a tundish and cast into a ribbon with a width of 50 mm and a thickness of approximately 0.5 mm under an argon atmosphere. The cast alloy was sectioned into 10 × 10 mm square metal sheets using a wire cutting machine. The samples were subjected to varying degrees of grinding and polishing using progressively finer sandpapers (the reciprocating friction treatment was conducted at room temperature (approximately 25 °C)): S80 (80# waterproof sandpaper), S400 (80#, 180#, and 400# sandpapers), S2000 (80#, 180#, 400#, 800#, 1000#, 1500#, and 2000# sandpapers), and SP (W2.5 diamond paste following S2000). During polishing, consistent conditions were maintained for the type, concentration, duration, speed, distribution, and pressure of the polishing agent. The polished samples were then etched in hydrofluoric acid (HF) solutions of varying concentrations (from 0.05 mol/L to 0.5 mol/L). Post-etching, the samples were sequentially washed with deionized water and anhydrous ethanol before being dried in a drying oven. The dried samples were prepared for further observation and analysis.

The microstructure of the differently roughened surfaces was examined using scanning electron microscopy (FEG-SEM, EHT = 15 kV, WD = 6.4 mm) (Zeiss, Oberkochen, Germany). The alloy’s phase composition was characterized by X-ray diffraction (XRD, D8 ADVANCE, Cu-Kα) (Bruker, Billerica, MA, USA), with a diffraction angle range of 20° to 80° and a scanning speed of 2°/min. Microhardness measurements were conducted on the samples with different surface finishes using loads of 100 N, 10 N, and 1 N to simulate macroscopic stress conditions.

## 3. Results

### 3.1. Wear Morphology of Amorphous Alloys and Microstructural Observation Post-Dealloying

**Figure 1:**
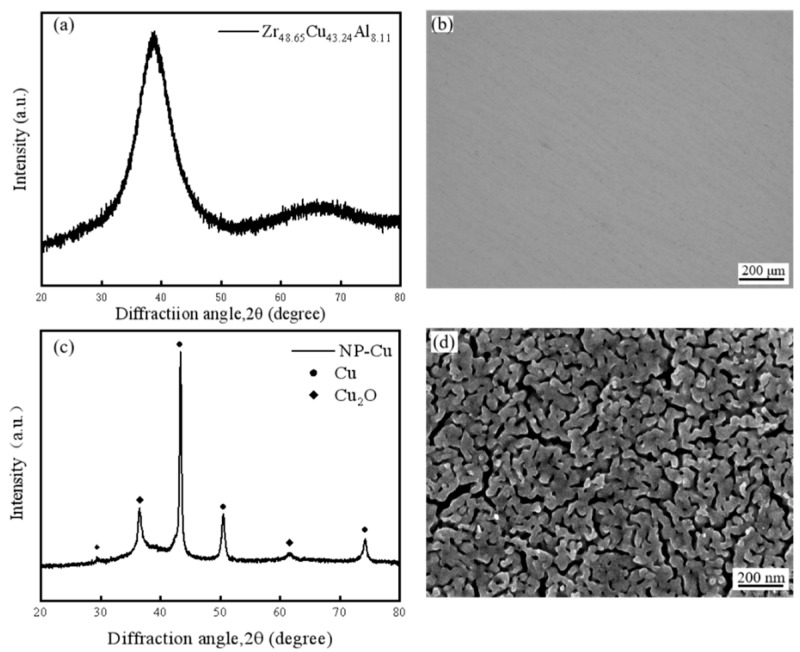
(**a**) XRD and (**b**) optical micrograph of the alloy, (**c**) XRD, and (**d**) SEM after dealloying.

As evidenced by the XRD test results depicted in Figure 1a, the spectrum exhibits an amorphous diffuse peak with no discernible crystalline diffraction peaks, indicating that the prepared alloy precursor is amorphous. This observation is corroborated by the optical microstructure image shown in Figure 1b, which reveals no crystalline phases in the matrix, further confirming the amorphous nature of the precursor. After dealloying, only Cu and oxygen are present on the sample surface, as indicated by the XRD measurements of Cu and Cu_2_O crystal peaks. Additionally, SEM images (Figure 1c,d) reveal a three-dimensional interconnected porous structure, confirming the formation of this structure post-dealloying.

**Figure 2:**
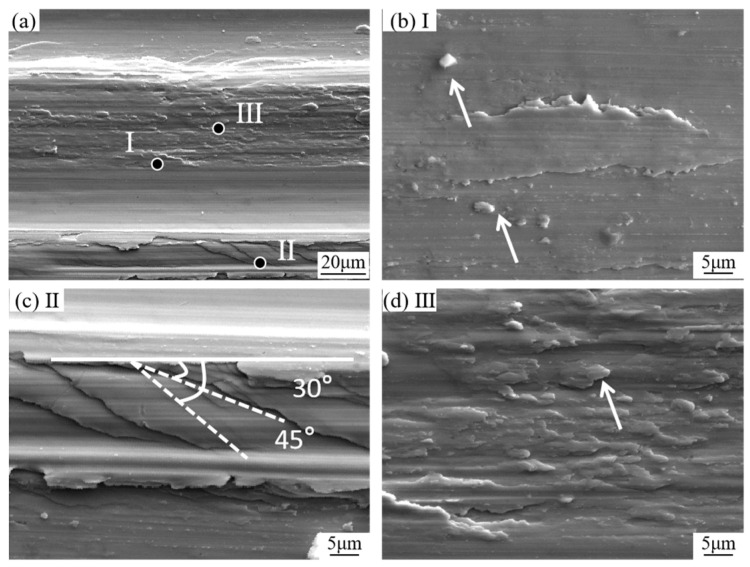
Morphology of different wear surfaces of amorphous alloy samples. In (**b**), the arrow indicates debris generated by abrasive wear, while in (**d**), it points to a river-like pattern formed during laminate peeling.

Figure 2a presents the SEM image of the sample surface post-rubbing. During the rubbing process, the amorphous surface layer undergoes significant changes, including furrows, edge shear zones, peeling pits, and surface material transfer. Figure 2b provides an enlarged view of point I, revealing distinct abrasive particles on the surface, which are formed by the crushing of flakes from the amorphous surface under high-friction stress [20]. As indicated in II of Figure 2c, shear bands can be observed, forming an angle of approximately 30 degrees with the rubbing direction. This observation supports the evidence that high shear stress induces non-uniform flow when the local temperature is below Tg during the rubbing process of amorphous materials (the contact temperature during reciprocating friction increased from room temperature (25 °C) to approximately 80–120 °C, depending on the wear time and applied load) [21]. The presence of tiny debris between friction pairs reduces the contact area, leading to very high local stress that may exceed the amorphous flow stress, thereby generating shear bands. Similar findings were reported by Liu [22], who also observed shear bands on the friction surface. Figure 2d illustrates the peeling of surface materials and river-like patterns due to the increase in contact temperature with wear time. When the temperature rises sufficiently, the amorphous alloy exhibits superplastic flow and adhesive wear, resulting in microfracture, peeling of the surface material, and material transfer, thus forming such morphologies.

**Figure 3:**
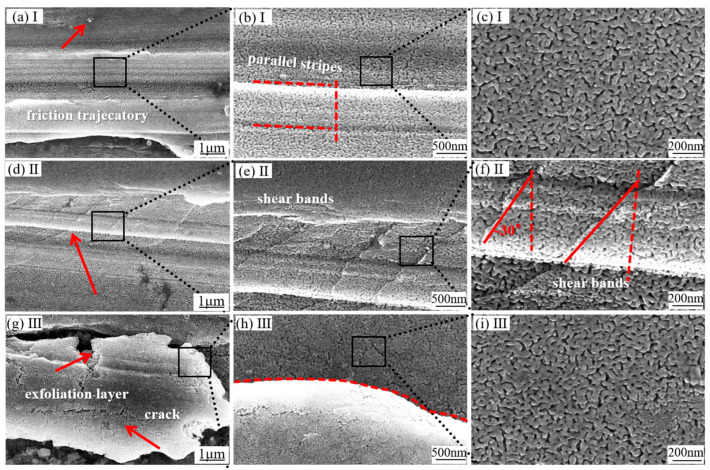
The SEM images in panels (**a**), (**d**), and (**g**) show different wear morphologies of amorphous alloy samples following dealloying treatment, with corresponding magnified views presented in (**b**,**c**), (**e**,**f**), and (**h**,**i**) respectively. (**a**) The arrow marks wear-induced debris formation. (**d**) highlights the development of shear bands during friction. (**g**) Indicates the boundary of layered peeling. The dashed lines in the images serve to enhance visualization of the structural features.

After dealloying, the sample retains some pre-dealloying wear morphology. As shown in Figure 3a, the number of abrasive particles on the surface decreases, but a wear track parallel to the friction direction remains visible. Figure 3b clearly shows the undulating surface of the dealloyed layer. High-magnification observations in Figure 3c reveal a uniform three-dimensional connected porous structure with consistent ligament and pore sizes. In the position depicted in Figure 3d, shear band stripes are still observable, though less pronounced than before corrosion, and the delamination phenomenon has disappeared. High-magnification examination of these stripes in Figure 3e,f indicates that they do not damage or disrupt the ligament or pore structure of NP-Cu, maintaining a uniform three-dimensional porous structure and good mechanical integrity. In Figure 3g, layered structures are observed attached to the matrix, and Figure 3h,i provides close-up views of the edges of these lamellar structures. The bottom dealloyed layer is well connected to the matrix, exhibiting good mechanical integrity and uniform ligament and hole structures. Measurements at 600 different points on the sample (excluding delamination positions) revealed pore sizes ranging from 6 to 30 nm, with an average pore size of 17 ± 3.12 nm. Ligament sizes ranged from 5 to 60 nm, with an average ligament size of 29 ± 6.56 nm.

### 3.2. Effect of Different Wear Degrees on NP-Cu Prepared by Dealloying

**Figure 4:**
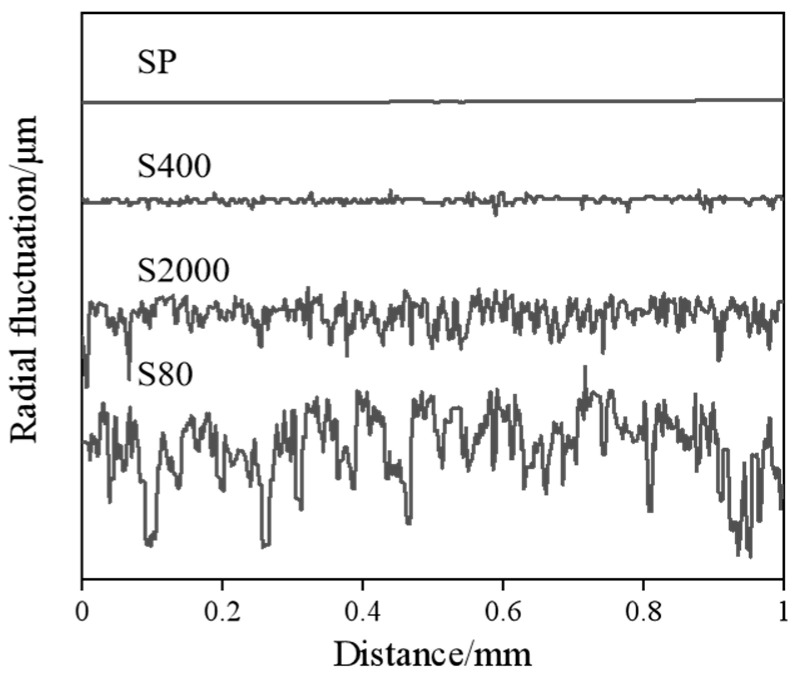
Profile of different friction surfaces of amorphous precursors.

According to the degree of friction and wear, the roughness of surfaces abraded by different sandpapers (S80, S400, S2000, and SP) was measured, yielding the arithmetic average roughness (Ra) values presented in Table 1. To more intuitively illustrate the similarities and differences among these surfaces, Figure 4 shows the profiles of the precursor’s different surfaces.

#### 3.2.1. NP-Cu Structure in Different Dealloying Environments

To minimize experimental error and the influence of dealloying process parameters, this section examines the effect of surface wear on NP-Cu preparation under varying dealloying conditions.

**Figure 5:**
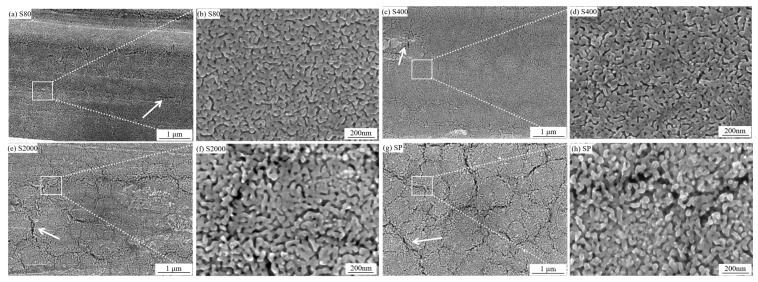
SEM images of S80, S400, S200, and SP with different roughness after dealloying. The arrow denotes the surface crack morphology in the figure.

When the etching solution concentration is 0.5 mol/L, the sample exhibits vigorous reactions, particularly on surfaces with higher roughness. After dealloying, distinct NP-Cu structures are observed on the S80, S400, S2000, and SP surfaces. Figure 5a,b shows the SEM image of the S80 surface post-dealloying. Parallel friction tracks are visible, with a few cracks (indicated by white arrows) appearing in areas of significant undulation. The overall NP-Cu structure remains well-preserved, and at higher magnification, micro-ligaments and pore sizes are evenly distributed. Figure 5c,d illustrates that the surface roughness of S400 is halved compared to S80, yet the surface structure remains largely intact. The depth of the friction grooves decreases, resulting in a flatter surface overall, with only a few porous cracks observable at high magnification. As shown in Figure 5e,f, the surface roughness of S2000 is one-tenth that of S80. At low magnification, a few network microcracks (white arrows) and uneven protrusions (white squares) are visible. At higher magnification, the pore structure becomes uneven, with porous cracks appearing. In Figure 5g,h, the surface roughness of SP is one-hundredth that of S80. At low magnification, numerous network-like cracks are observed in the dealloyed layer, while at high magnification, ligament fractures and uneven pore sizes are evident.

**Figure 6:**
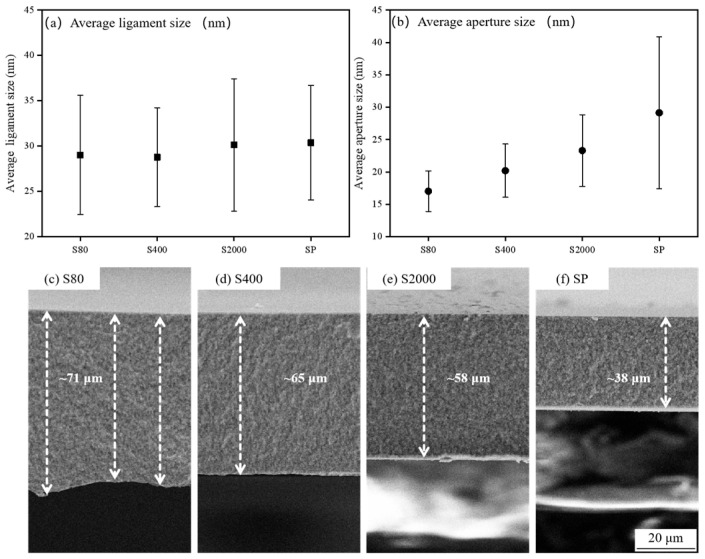
(**a**,**b**) A comparative analysis of the average ligament size and aperture size; (**c**–**f**) A comparison of NP-Cu layer thickness following the dealloying of different surfaces: S80, S400, S2000, and SP.

Figure 6a,b compares the ligament and pore sizes across different NP-Cu structures. According to the statistical results, as surface roughness decreases, the ligament size remains relatively unchanged, but the pore size increases and its distribution becomes more uneven. Figure 6c–f compares the thickness of the dealloyed layer on four different surfaces, showing that the thickness decreases with decreasing roughness. When roughness decreases by an order of magnitude (from S80 to S2000 and SP), the thickness decreases by approximately 20 μm.

To further reduce the influence of dealloying process parameters and provide a clearer comparison of the effect of roughness on dealloying thickness, the thickness contrast between S80 rough and SP smooth surfaces was calculated under identical concentrations, different dealloying times, and different concentrations at the same time. As shown in Table 2, at the same concentration, the thickness of the dealloyed layer increases with time. After 30 min of dealloying, the thickness of the S80 and SP layers are nearly equal, but after 24 h, the thickness of S80 is nearly four times that of SP. To enhance experimental accuracy, specific surface area measurements were conducted under the same conditions. After 2 h of dealloying at a 0.5 mol/L corrosion concentration, the specific surface area of S80 was 14.419 m^2^/g, while that of SP was 7.994 m^2^/g. Table 3 indicates that at the same dealloying time, increasing the concentration leads to more pronounced changes in the thickness of the dealloyed layer. Although the thickness of the dealloyed layer on rough surfaces remains greater than that on smooth surfaces, the influence of concentration becomes more significant as it increases.

#### 3.2.2. Study on Mechanical Behavior of Different Rough Surfaces

Due to the brittleness of the amorphous alloy layer and the nano-porous structure of the dealloyed layer in the composite materials, these materials are highly susceptible to stress concentration. Mechanical tests, such as tensile or cyclic stress tests, typically result in fractures occurring within the amorphous alloy layer rather than at the NP-Cu interface. This limitation hinders the investigation of adhesion and structural integrity at the NP-Cu interface. To address these challenges and maintain scientific rigor, we employed a testing approach that combines hardness testing and structural morphology analysis. This method allowed for an indirect yet quantitative assessment of the mechanical properties of the NP-Cu layer and its adhesion to the underlying substrate.

**Figure 7:**
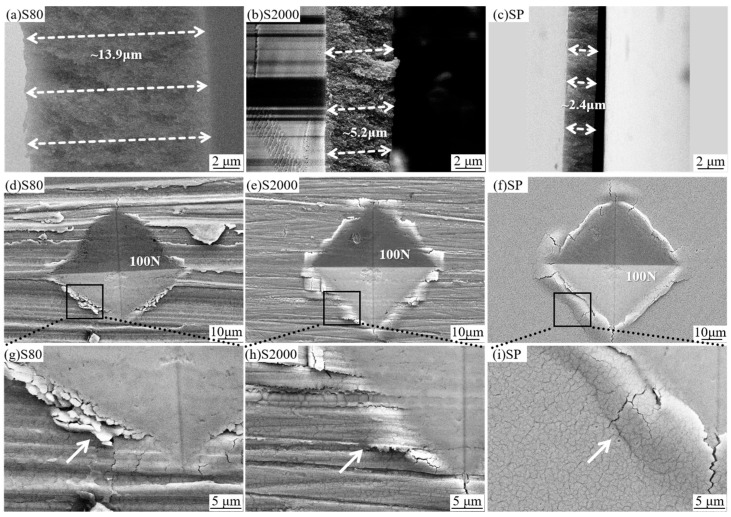
Microstructural characterization of dealloyed layers on surfaces with varying roughness: (**a**–**c**) Cross-sectional SEM images showing the thickness variation of dealloyed layers on (**a**) S80, (**b**) S2000, and (**c**) SP surfaces; (**d**–**f**) Surface SEM morphologies of hardness indents formed during dealloying of NP-Cu under 100 N loading on (**d**) S80, (**e**) S2000, and (**f**) SP surfaces; (**g**–**i**) High-magnification SEM images of the corresponding indents in (**d**–**f**).

A force of 100 N was applied to the dealloyed layers on different surfaces, and the surface state was observed, structural changes were analyzed, and mechanical behaviors were evaluated by combining hardness values. The dealloying experiment was carried out in a low-concentration corrosive environment. It is easier to prepare nano-porous structures with excellent mechanical integrity by dealloying in a low-concentration environment [23]. Figure 7a–c illustrates the thickness of the dealloyed layers for S80, S400, S2000, and SP. As the roughness decreases, the thickness of the dealloyed layers is reduced by approximately half. Figure 7d–f depicts the morphologies of S80, S2000, and SP surfaces under a 100 N hard-point indentation. Notably, the indentations on S2000 and SP exhibit rhomboidal shapes with equal diagonal lines, whereas the diagonal rubbing direction of the rhomboid indentation on S80 is longer than its vertical direction. With decreasing roughness, more cracks appear around the hardness point. Figure 7g, a partial enlargement of (d), shows that at S80, numerous stacked NP-Cu fragments surround the hardness point, with few cracks or raised areas. In contrast, the enlarged view of Figure 7i clearly reveals cracks caused by forced extrusion, where the dealloyed layer at the edge of the indentation bulges and separates from the matrix layer, compromising structural integrity. This phenomenon becomes more pronounced as the surface smoothness increases. Morphological analysis indicates that under identical stress conditions, the hardness pit on rougher surfaces is smaller compared to smoother surfaces, suggesting that the dealloyed layer on rough surfaces exhibits greater deformation resistance and is less prone to microcracks.

**Figure 8:**
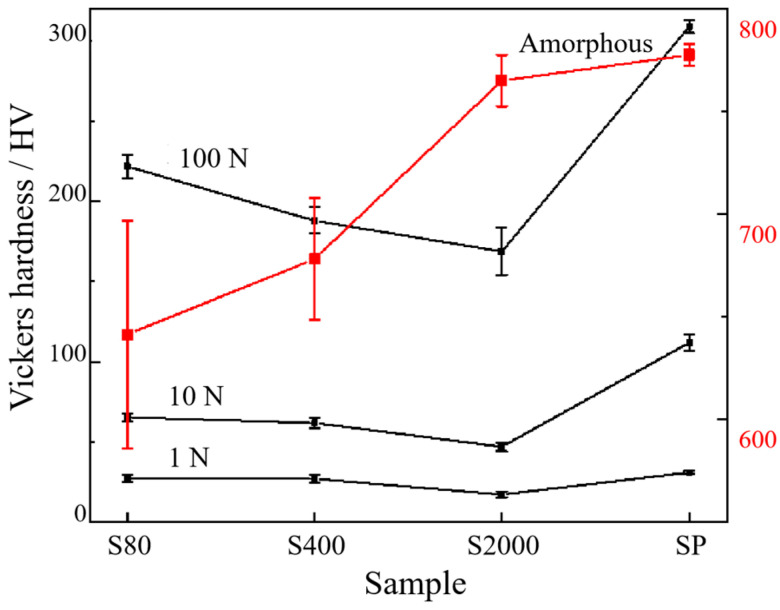
Average change in four kinds of surface hardness values under different forces.

Forces of 100 N, 10 N, and 1 N were applied to the surfaces of dealloyed amorphous precursors, S80, S2000, and SP, respectively, to obtain hardness values at different forces and compare stress behaviors across varying roughness levels. As shown in Figure 8, all three curves initially decrease and then increase. Within a certain roughness range, hardness values decrease with decreasing roughness. However, when the surface reaches a specific level of smoothness, hardness values significantly increase. For instance, under the same force, the surface hardness value of SP is higher than that of other surfaces, and this increase is more pronounced with greater force. This change in hardness values correlates with the thickness of the dealloyed layer.

## 4. Discussion

### 4.1. Analysis of the Influence of Surface Wear Mechanism During NP-Cu Preparation by Dealloying

The wear mechanism during the preparation of nanoporous copper (NP-Cu) is inherently complex, involving adhesive wear, abrasive wear, fatigue wear, and corrosion wear, which may act independently or synergistically to influence the material’s surface morphology, structure, and properties [24,25]. During friction, the force exerted by micro-convexities on the precursor can be resolved into tangential and normal components. At an appropriate angle, the normal force presses the micro-convexity into the surface, while the tangential force cuts the surface, forming directional friction tracks, as illustrated in Figure 2. Due to varying contact angles, microprotrusions push the surface material forward and sideways, causing plastic deformation and forming parallel shear bands (Figure 2c).

Adhesive wear further contributes to material migration and the formation of microcracks at the interfaces between laminae and the matrix, leading to ligament fractures and the creation of tiny corrosion pits on the NP-Cu surface. These pits play a critical role in the subsequent dealloying process as they evolve into parts of the nanoporous structure, optimizing pore distribution and morphology. This synergistic interplay between wear mechanisms not only enhances surface roughness but also facilitates the formation of a well-defined nanoporous architecture, ultimately improving the structural integrity and functionality of the NP-Cu layer.

Increased roughness enhances the equilibrium constant of the dealloying corrosion reaction [26,27], providing a larger specific surface area and energy, which promotes stronger surface effects, offers more active sites for corrosion reactions, accelerates the dealloying process, thickens the prepared NP-Cu layer, improves NP-Cu surface activity, and facilitates reactant adsorption and transformation [28,29]. Furthermore, surface roughness plays a critical role in the dealloying process by influencing the removal rate of Cu atoms. Rough surfaces, with their higher density of defects and asperities, create localized stress concentrations that facilitate the preferential dissolution of Cu atoms during dealloying. This enhanced dissolution is driven by the increased reactivity of rough surfaces, which accelerates the breakdown of atomic bonds and promotes the selective removal of Cu atoms from the precursor alloy. As a result, the dealloying process becomes more efficient, leading to a more uniform and well-defined nanoporous structure. The interplay between surface roughness and dealloying kinetics underscores the importance of controlling surface morphology to optimize the fabrication of NP-Cu with desired properties.

However, severe wear can introduce significant defects such as scratches, pits, and peeling on the precursor alloy film surface, disrupting material continuity and affecting the uniformity and integrity of nanoporous structures.

### 4.2. Analysis of the Influence of Surface Roughness on NP-Cu Structure and Stress Process

According to the literature [30,31], the surface energy of rough surfaces is generally higher than that of smooth surfaces, which promotes atomic migration and enhances chemical reaction rates. In addition, surface defects and localized stress concentrations significantly accelerate the etching process. Friction increases the number of surface defects, thereby indirectly enhancing the etching rate. Meanwhile, rough surfaces provide more diffusion pathways and exhibit higher atomic mobility. Based on the above analysis, friction theoretically plays a promoting role in the dealloying process. (It should be noted that since the temperature of the amorphous alloy surface during friction does not reach the critical value required for crystallization, the influence of grain boundaries on the dealloying process is not discussed in this study.) This accelerates the corrosion reaction, leading to thicker nanoporous carbon (NP-Cu) layers under identical dealloying conditions.

**Figure 9:**
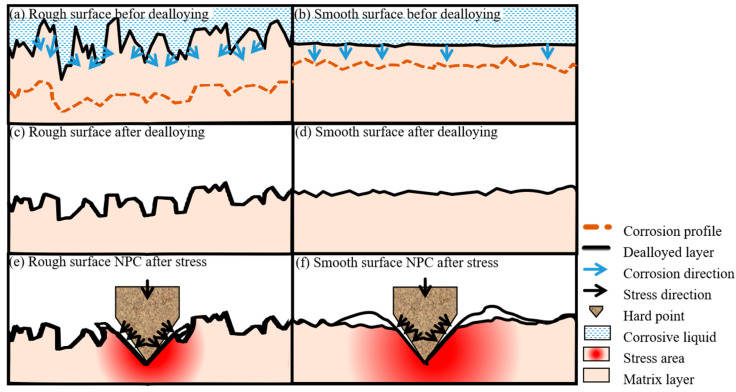
(**a**,**b**) Schematic diagram of rough surface and smooth surface before dealloying; (**c**,**d**) schematic diagram of dealloyed surface; (**e**,**f**) schematic diagram of surface dealloyed layer when different surfaces are stressed.

As illustrated in Figure 9a,b, surfaces with higher roughness form microscale closed spaces during corrosion. The humidity, temperature, and chemical composition within these spaces differ from the external environment, resulting in varying corrosion rates at different positions. Corrosion products may accumulate in depressions, providing partial protection to the underlying substrate from further corrosion. Under various factors, the dealloyed layer on rough surfaces tends to become more uniform. In contrast, on smooth surfaces, corrosion primarily occurs at vulnerable points, and corrosion products are easily washed away or fall off, leading to continuous inward corrosion of the substrate and forming uneven morphologies after dealloying. Consequently, there is a significant difference in microstructure and flatness between dealloyed surfaces with varying degrees of roughness, as shown in Figure 9c,d.

Numerous studies have demonstrated that the compressive strength of nanoporous structures is influenced by ligament link strength and pore size [32,33,34]. Structures with higher ligament connection strength and smaller pore sizes exhibit better deformation resistance. During dealloying, volume shrinkage and stress concentration can induce residual stress in the precursor alloy film. Appropriate surface wear can help release these residual stresses, thereby reducing their adverse effects on the integrity and performance of nanoporous structures and enhancing structural stability. As depicted in Figure 9e,f, when rough surfaces are subjected to stress, scratches on the contact surface reduce the stress concentration under high loads, minimize the affected area, inhibit crack initiation and propagation, and decrease damage to the NP-Cu structure, thus improving its deformation resistance. Smooth surfaces exhibit opposite behavior.

In Figure 8, the hardness of samples increases slightly under different stress levels, with this increase becoming more pronounced as stress increases. This phenomenon is attributed to the varying thicknesses of the dealloyed layers on different surfaces. The stress experienced by the sample is a combined effect of the dealloyed layer and the matrix layer. For SP samples with thinner dealloyed layers, the primary stress-bearing component is the amorphous matrix, which has higher deformation resistance, leading to higher hardness values. As the thickness of the dealloyed layer increases, its proportion in the overall structure rises, resulting in an increase in hardness. The red line in the figure represents the hardness change in the amorphous matrix under a 100 N load. At this point, the average hardness value of the S80 rough surface exceeds that of the amorphous matrix, indicating that the dealloyed layer bears a greater proportion of the stress. With decreasing roughness, this gap narrows, suggesting a reduction in the thickness of the dealloyed layer and a shift in stress distribution towards the amorphous matrix, leading to a decrease and then an increase in hardness. This process confirms that the dealloyed layer possesses deformation resistance, with higher surface roughness correlating with thicker dealloyed layers and stronger resistance.

## 5. Conclusions

Surface wear exhibits a dual-edged effect during the preparation of nanoporous copper (NP-Cu) via dealloying. While excessive wear can introduce adverse effects such as surface damage, contamination, and property degradation, moderate friction and wear positively influence the dealloying process. Controlled friction generates precursor surfaces with tailored roughness, which removes surface oxides and exposes fresh alloy surfaces. This facilitates uniform penetration of the corrosive medium, promoting the formation of a homogeneous nanoporous structure. Additionally, friction-induced plastic deformations modify the alloy’s microstructure, enhancing the formation and propagation of corrosion channels during dealloying. However, these benefits are limited to specific conditions, as excessive wear can severely compromise surface integrity and material properties. Therefore, optimizing friction levels and surface roughness is critical for achieving high-quality NP-Cu structures.

In summary, controlled surface wear is a promising strategy for enhancing NP-Cu preparation. However, the interplay between wear mechanisms and dealloying is complex and requires further investigation. Future research should focus on: (1) mechanistic studies to understand the effects of wear on microstructure evolution, stress distribution, and catalytic performance; (2) establishing quantitative relationships between surface roughness and NP-Cu properties to optimize preparation processes; (3) developing precision control methods for surface roughness through advanced fabrication techniques; and (4) material optimization by addressing the macro-fragility of NP-Cu and exploring amorphous alloys as precursors for other nanoporous metals.

This study not only advances the fundamental understanding of friction-assisted dealloying but also provides practical insights for improving NP-Cu applications in catalysis, sensors, and electronics. By addressing these challenges, we aim to broaden the scope of nanoporous material fabrication and inspire further innovations in the field.

## Figures and Tables

**Table 1:** Ra of S80, S400, S2000, and SP.

Sample	S80	S400	S2000	SP
R_a_	0.808 ± 0.0356	0.3426 ± 0.0148	0.01836 ± 0.001228	0.00156 ± 0.0002

**Table 2:** Thickness values of dealloyed layers of S80 and SP after dealloying at different times when the concentration of corrosive solution is 0.5 mol/L.

Sample	30 min	1 h	1.5 h	2 h	24 h
S80	21.9 μm	48.5 μm	58.7 μm	71.4 μm	496.2 μm
SP	20.4 μm	30.3 μm	37.0 μm	38.3 μm	134.1 μm

**Table 3:** Thickness values of dealloyed layers of S80 and SP after dealloying with different corrosion concentrations when the etching solution time is 2 h.

Sample	0.005 mol/L	0.05 mol/L	0.5 mol/L
S80	5.8 μm	15 μm	71.4 μm
SP	1.3 μm	2.9 μm	38.3 μm

## Data Availability

The original contributions presented in this study are included in the article. Further inquiries can be directed to the corresponding author.
